# Psychometric assessment of HIV/STI sexual risk scale among MSM: A Rasch model approach

**DOI:** 10.1186/1471-2458-11-763

**Published:** 2011-10-05

**Authors:** Jian Li, Hongjie Liu, Hui Liu, Tiejian Feng, Yumao Cai

**Affiliations:** 1 National Center for AIDS/STD Control and Prevention, Chinese Center for Disease Control and Prevention, Beijing, China; 2 Department of Epidemiology and Community Health, School of Medicine, Virginia Commonwealth University, Richmond, Virginia, USA; 3 Department of HIV/STD Control and Prevention, Shenzhen Center for Chronic Disease Control, Shenzhen, China

## Abstract

**Background:**

Little research has assessed the degree of severity and ordering of different types of sexual behaviors for HIV/STI infection in a measurement scale. The purpose of this study was to apply the Rasch model on psychometric assessment of an HIV/STI sexual risk scale among men who have sex with men (MSM).

**Methods:**

A cross-sectional study using respondent driven sampling was conducted among 351 MSM in Shenzhen, China. The Rasch model was used to examine the psychometric properties of an HIV/STI sexual risk scale including nine types of sexual behaviors.

**Results:**

The Rasch analysis of the nine items met the unidimensionality and local independence assumption. Although the person reliability was low at 0.35, the item reliability was high at 0.99. The fit statistics provided acceptable infit and outfit values. Item difficulty invariance analysis showed that the item estimates of the risk behavior items were invariant (within error).

**Conclusions:**

The findings suggest that the Rasch model can be utilized for measuring the level of sexual risk for HIV/STI infection as a single latent construct and for establishing the relative degree of severity of each type of sexual behavior in HIV/STI transmission and acquisition among MSM. The measurement scale provides a useful measurement tool to inform, design and evaluate behavioral interventions for HIV/STI infection among MSM.

## Background

Sexual risk behavior is a major determinant of HIV and STI transmission or acquisition for men who have sex with men (MSM) and their sexual partners. Effective HIV/STI intervention and prevention relies on better understanding and measurement of sexual risk behaviors. In general, there are two methods that are commonly used to measure the degree of sexual risk for HIV/STI infection among MSM. The first method is to create binary or categorical variables to measure the frequency of different types of risky sexual behaviors (items) [1]. For example, subjects are asked how often they used condoms when practicing anal sex with MSM in the last three months, or used condoms when practicing oral sex with male sex workers in the last three months. While this method is easily implemented, it lacks sensitivity in measuring sexual risk since a single measure can only cover a limited domain of sexual risk for HIV/STI infection [2]. Moreover, it lacks statistical power in the analyses of correlates of a certain sexual behavior since researchers usually treat each type of risky sexual behavior as a single binary or categorical variable in univariate and multiple analyses.

The second common method to measure sexual risk is to sum the number of risky sexual behaviors (items) to form an ordinal index with a summary composite score [3]. This index is presumed to cover a continuum ranging from no sexual risk (a score of 0) to many types of sexual risk (a score of 1 or more). In such a scale, all items that indicate different types of sexual behaviors contribute equally to the total score, and no consideration is given to the degree of severity of an item that reflects a particular type of sexual behavior. For example, having unprotected anal sex with male sex workers would contribute 1 point to the total score, the same as having unprotected oral sex with regular sex partners. The former behavior is likely to represent a far greater level of sexual risk for HIV/STI infection than the latter, yet this would not be reflected in the total score. Because of the ordinal nature of an index, the difference between two scores may have different levels of sexual risk at different parts of the continuum. For example, the difference between scores of two to four may not be the same as the same difference between scores of five to seven, in terms of the degree of severity of sexual risk for HIV/STI infection.

Although the index approach is commonly used in research, the degree of severity of an individual's sexual risk for HIV/STI infection may not be represented adequately when indexed solely by the frequencies of sexual behaviors reported. The degree of risk severity also is related to the particular types of sexual behaviors that the individual engages in. Evaluation of the likelihood of a specific type of sexual behavior as a function of the overall level of sexual risk provides information about the degree of risk severity of a particular behavior in relation to one another, the ordering and pattern of risky sexual behaviors, and the magnitude of differences reflected by endorsement of additional risky behaviors [4]. This detailed information can increase researchers' understanding of the composition and degree of sexual risk for HIV/STI infection by sexual behaviors.

## The Rasch model

To overcome the limitations of these two measurement methods, we used the Rasch model to assess the degree of severity of sexual behaviors for HIV/STI infection. The Rasch model was named after the Danish mathematician Georg Rasch [5]. Although the model has been widely used in education research, the application of the model in research of HIV/STI-related sexual risk is scant [6]. The Rasch model measures the relationship between a person's ability and an item difficulty, and models this as a probabilistic function. Specifically, raw data from a rating scale is converted to "an equal interval scale" measured in logits (log odd units), reflecting the item difficulty and person's ability [7,8]. In this study, the person's ability refers to the level of sexual risk that a person possesses, and the item difficulty refers to the level of risk for HIV/STI infection that is associated with the behavior (the item). In the Rasch model, the probability of a certain sexual behavior that is engaged in by a person depends on this person's ability (proficiency in engagement in a risk behavior) relative to the difficulty of this item (risk for disease transmission). If the data fit the Rasch model well, the model transforms an ordinal raw composite score into a linear, interval-level variable. The transformed scale is on an interval scale in the unit of logit. A greater logit value for an item indicates increasing item difficulty; a person with a higher logit value possesses a higher degree of risk severity of sexual risk than a person at a lower level.

In this study, an HIV/STI sexual risk scale was developed using data on sexual behaviors obtained from MSM in China. The scale included items related to unprotected intercourse, including non-commercial sex, commercial sex, and concurrent sexual partnerships. Psychometric properties of the scale were assessed in a dichotomous Rasch model. Findings of the Rasch model analysis were used to answer the following questions of primary interest: (1) to what extent do the items of the scale measure a single dimension of the level of sexual risk? (2) what is the relative severity and ordering of the different types of sexual behaviors in the scale?

## Methods

### Participants

The detailed procedures of the recruitment of study subjects have been previously described [9]. Briefly, a cross-sectional study of social network factors associated with HIV/STI-related risks was conducted among MSM in Shenzhen, China. A man was eligible for this study if he: (1) was between 18-45 years old; (2) reported having engaged in anal intercourse with one or more men in the past year; and (3) had lived in Shenzhen for more than three months at the time of the interview. Respondent-driven sampling (RDS) was used to recruit MSM [10]. A group of 12 seeds were selected and each was given three coded recruitment coupons to refer up to three peers from their networks. These seeds were heterogeneous in age (18-30 and 31-45 years old), MSM congregation venue (sauna, bar, and public park), and engagement in commercial sex (engaged vs. not engaged). The planned sample size of 351 eligible MSM was obtained after four to five waves of RDS recruitment. Eligible MSM recruited by the 12 seeds at wave one and new recruits at subsequent waves participated in a face-to-face anonymous interview in a private interview room. The study protocol was approved by the Institutional Review Boards of the Virginia Commonwealth University and Chinese Center for AIDS/STD Control and Prevention.

### Measures

The HIV/STI sexual risk scale included nine forms of sexual behaviors (9 items), including items related to unprotected intercourse, participating in non-commercial sex, commercial sex and concurrent sexual partnerships. Respondents were asked how often they used condoms in each of eight sexual behaviors in the past six months: oral sex with MSM, anal sex with MSM, anal sex with male sex workers, oral sex with male sex workers, anal sex with male clients, oral sex with male clients, sex with female sex workers, and sex with regular partners (wife or girlfriend). Responses to these eight items were in the forms of frequencies from 'use every time' to 'never use'. Count data were recoded into dichotomies (0 = safer sex: used condoms in every sexual act; 1 = unsafe sex: not used condoms for every sexual act). Concurrent sexual partnerships were assessed by comparing dates of first and last sexual intercourse with sex partners in the past six months. Overlapping of these dates was categorized as "had concurrent partnerships". Some potential dependencies might exist in the data. For example, an endorsement of either item four or item six would lead to the endorsement of item one. These items were not altered in order to allow the Rasch residual analyses determine if the degree of local dependence was enough to warrant post-hoc recoding of the data [6]. All the items are listed in Table 1.

**Table 1:** Original nine items on the HIV/STI sexual risk scale

Item	Item content	Frequency	Percent (%)
Item 1	Had unsafe oral sex with MSM	265	75.5
Item 2	Had unsafe anal sex with MSM	139	39.6
Item 3	Had unsafe anal sex with male sex workers	6	1.7
Item 4	Had unsafe oral sex with male sex workers	23	6.6
Item 5	Had unsafe anal sex with male clients	15	4.3
Item 6	Had unsafe oral sex with male clients	40	11.4
Item 7	Had unsafe sex with female sex workers	5	1.4
Item 8	Had unsafe sex with regular sex partners	62	17.7
Item 9	Had concurrent sex partners	208	59.3

### Statistical analysis

Using WINSTEPS (version 3.68.2), the Rasch model analysis was carried out to examine how well the observed data fit the expectations of the measurement model.

#### Reliability

Person-reliability and item-reliability are the major measures of reliability that are given by fitting the Rasch model. Person-reliability is equivalent to the traditional test reliability [11], which indicates how likely we will be able to get the same ordering of individuals using a repeated test [7]. High person-reliability means that we have developed a line of inquiry in which some persons score higher and some score lower, and that we could expect consistency of these inferences [7]. Item-reliability refers to the ability of the test of define a distinct hierarchy of items along the measured variable on a 0 to 1 scale. The higher the number, the more confidence we can place in the replicability of item placement across other samples [7].

#### Unidimensionality and local independence

The dichotomous Rasch model was the basic method used for the item analyses, which was appropriate for two ordered categories scoring structure [7]. The important assumptions of the dichotomous Rasch model are unidimensionality and local independence. Unidimensionality means that a single construct (the latent trait) is being measured by a set of items (e.g., different types of sexual behaviors). Local independence means that the entire correlation between the items has to be captured by the latent trait. Correlations between the items that are not accounted for by the latent trait (i.e. the person parameter) are indicative of local dependence which may be a cause for concerns, reflecting either multidimensionality or response dependence [12,13]. The Principle Components Analysis (PCA) of standardized residuals was applied to analyze item dimensionality and local independence. WINSTEPS does a PCA of residuals, not of the original observations. Therefore, the first component (dimension) had already been removed. So we could analyze secondary dimensions, components or contrasts. Despite some exceptions, guidelines for assessing unidimensionality via PCA include the following: variance explained by items greater than four times the first contrast is good; variance explained by measures greater than 50% is good; and unexplained variance explained by first contrast (eigenvalue size) less than 3.0 is good [11]. Local independence between items was appraised by inspecting the largest residual correlations for pairs of items with correlations with absolute values less than 0.30 [14,15].

#### Rasch Fit Statistics

Once the parameters of a Rasch model are estimated using the maximum likelihood estimation process, they are then used to compute expected responses of each person to every item. Fit statistics are then derived from a comparison of the expected and observed responses. These "fit statistics" may be used to detect departures from the Rasch model requirements of unidimensionality and items that may be statistically dependent with other items.

WINSTEPS provides two types of fit statistics for persons and items [11]. Infit is an information-weighted fit statistic which is more sensitive to unexpected behavior affecting responses to items near the person's measure level of sexual risk. Outfit is an outlier-sensitive fit statistic, more sensitive to unexpected behavior by persons on items far from the person's measure level of sexual risk [11]. Unstandardized fit estimates (mean square) are modeled by the Rasch algorithm to have a mean of 1. With the mean closer to the expected 1, the infit mean squares show less spread from the ideal and outfit mean squares show greater variation. The standardization of fit scores has an approximate *t *distribution with mean of 0 and a standard deviation near 1. Negative values indicate less variation than modeled. Positive values indicate more variation than modeled. Infit and outfit *t *values greater than +2 or less than -2 generally are interpreted as having less compatibility with the model than expected (*p *< 0.05) [7]. Values greater than +3 will be used to identify items for further review [6]. Because the infit statistic gives relatively more weight to the performance of persons closer to the item value, infit values will be more closely scrutinized than outfit values in our study [7].

An item-person map was produced by WINSTEPS software, in which the items were indicated by the item numbers and an individual person's performance were represented by "#". The relationship between item difficulty and person ability was very clear by the data represented in the Rasch variable map format.

#### Item difficulty invariance

Rasch developed a unidimensional measurement model that reflects the basic criterion of invariance which is a crucial feature of fundamental measurement [16]. The invariance criterion means that an instrument, in principle, is required to work in the same way for all individuals, e.g. whether the items worked in the same way for subjects with high sexual risk as for those with low risk. In order to test item difficulty invariance, subjects were divided in two groups according to their ability. The difficulty estimates of items from each of the analyses with the high ability and low ability subsamples were plotted onto a simple scatter plot on the corresponding *x *and *y *axes by using the Rasch-modeled ability estimate measures (in logits) for each item. Quality control lines for a 95% confidence band were used to see whether the distribution of the plotted ability points were close enough to the modeled relationship diagonal line for the measures to be regarded as sufficiently invariant [7].

## Results

### Description of the study sample

In total, 351 eligible subjects were recruited and interviewed. Age ranged from 18 to 44 years of age (mean: 27 years old; standard deviation: 6). More than half of the subjects (65%) received a high-school education or above and 5% received either only a primary school education or no education at all. More than two-thirds (78%) were single. Thirty-nine percent of MSM worked in entertainment venues, such as bars, saunas, night clubs or dance halls. Among 351 MSM, 58 (17%) were Money boys ("MBs"; i.e. males who sell sex to MSM). There were 92 (26%) MSM who had ever engaged in commercial sex in the past six months, including 58 (17%) selling sex to MSM, 34 (10%) buying sex from MBs, and 19 (5%) buying sex from female sex workers.

### Reliability

**Figure 1:**
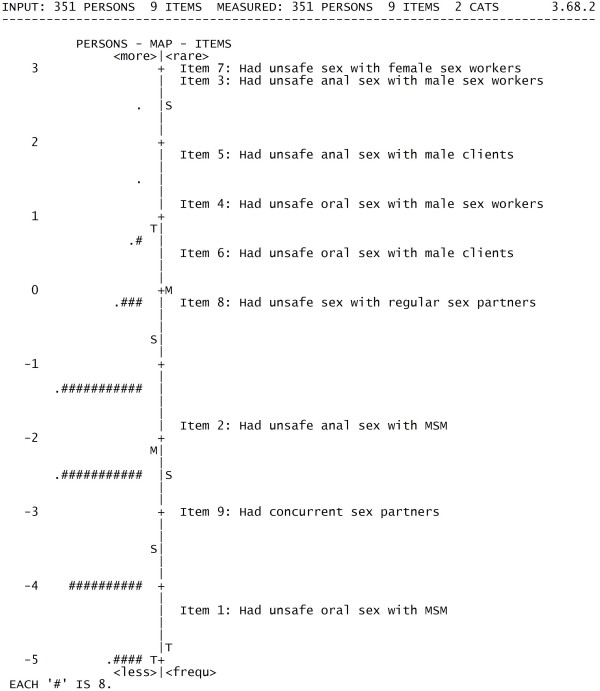
**Wright map**.

The person reliability estimated in Rasch model is 0.35. The item reliability is 0.99. The low person reliability indicated that the sample had a narrow spread in items [17], i.e., the majority of subjects engaged in unsafe sex measured in item 1, 2 and 9, which is consistent with the results from Table 1 and the Wright map (Figure 1). If a majority of subjects had reported engaging in both relatively high and low risk sexual behaviors, the person reliability would be higher. The person reliability can also be increased by adding people who have high or low scores to our sample, since the greater the sample variance on the dimension being measured, the greater the person reliability. The level of HIV/STI sexual risk behaviors in this sample was relatively low. However, a scale including relative high risk sexual behavior items compared to the target population was necessary because no risk sexual behavior was considered too high in HIV/STI prevention and intervention. The high item reliability indicated that this scale included items measuring sexual risk from the lower level to higher level. Because the Rasch model overcomes the major limitation of True Score Theory, which is the sample dependency of item and test indices and the item dependency of person ability [18], we can proceed with the analysis since the scale had a very high reliability in measuring the intended behaviors.

### Unidimensionality and local independence

The analysis of standardized residuals in PCA indicates that the Rasch dimension explained 55.4% of the variance in the data. It was slightly above the guidelines for assessing unidimensionality via PCA (50%), which was relatively low compared with 97.6% from a study on risk behavior scales [6], and was similar to the one (61%) reported from a study of the 8-item Parkinson's Disease Questionnaire [19]. The largest secondary dimension (the first contrast in the residuals) explained 7.7%. The variance explained by the items (42.1%) was more than five times the variance explained by the first contrast (7.7%). The eigenvalue of the first contrast was 1.5. The largest standardized residual correlations used to identify dependent items were from -0.34 to 0.31. Two pairs of items had absolute values of correlations greater than 0.30: 0.31 for "Had unsafe anal sex with male sex workers" and "Had unsafe oral sex with male sex workers"; -0.34 for "Had unsafe anal sex with MSM" and "Had concurrent sexual partner". A positive residual correlation between the two items was expected because the MSM who spent money on sex might want to make full use of this investment by enjoying greater sexual intimacy and physical stimulation [20]. The negative correlation between the other two items indicates that MSM reporting concurrent sexual partnerships may be more likely to report condom use [21]. Although the residual correlations occurred, we decided to retain these two pairs of items and not to combine them because they measured different severity of sexual risk and these residual correlations were marginal to the criterion value of 0.30, indicating that the reliability was unlikely to be adversely inflated [14].

### Fit statistics

For items, the unstandardized fit estimates (mean square) were 0.98 for infit and 1.03 for outfit, the standardized fit estimates (ZSTD) are 0.10 for infit and 0.20 for outfit. For persons, the unstandardized fit estimates (mean square) were 1.01 for infit and 0.81 for outfit, the standardized fit estimates (ZSTD) for both infit and outfit are 0.

Table 2 presents the estimate of item difficulty of each item and its accompanying error estimate in logits. Fit statistics are shown in their natural (mean square) and standardized forms (standardized as *t*). Item7, "Had unsafe sex with female sex workers", was the most difficult item. Item 1, "Had unsafe oral sex with MSM", was the easiest item. According to the infit *t *values, all the items fit the model well except item 2 with an infit *t *value of 2.40, which is still less than +3. Fit statistics of person ability indicate that only eight subjects' infit *t *values were beyond the range of -2 and +2 and only two subjects' values were above +3 among 351 subjects.

**Table 2:** Item difficulty estimates with associated error estimates for each item

Item	Difficulty Estimate	Error Estimate	Infit Mean Square	Outfit Mean Square	Infit t	Outfit t
Item 1	-4.36	0.17	0.99	1.98	-0.10	2.90
Item 2	-1.80	0.13	1.15	1.18	2.40	1.70
Item 3	2.85	0.43	0.89	1.48	-0.20	0.90
Item 4	1.24	0.24	1.05	0.77	0.30	-0.40
Item 5	1.79	0.29	0.73	0.28	-1.30	-1.60
Item 6	0.48	0.19	0.95	0.71	-0.40	-0.90
Item 7	3.06	0.47	1.08	0.82	0.30	-0.10
Item 8	-0.20	0.16	1.08	1.31	0.80	1.40
Item 9	-3.06	0.14	0.91	0.77	-1.30	-1.60

The item-person map (Figure 1), called a Wright map, depicts the item difficulty and person ability. The vertical line represents the measure of severity of sexual risk, with logit values given on the left. Persons' ability levels are presented as "#" symbols and aligned to the left of the corresponding measure. The M, S, and T on each side of the vertical line separating the person and item distributions represent the mean, one standard deviation and two standard deviations. Persons with higher risk behavior and items that are more difficult to endorse are distributed closer to the top of the figure. According to the Wright map, items are distributed evenly from the top to the bottom. Item 7 was the most difficult to endorse and item 1 was the easiest one, which was same with the results of item difficulty estimates. Most persons distributed below 0 logit value, which meant the items were too difficult for the people included in the study.

### Item difficulty invariance

**Figure 2:**
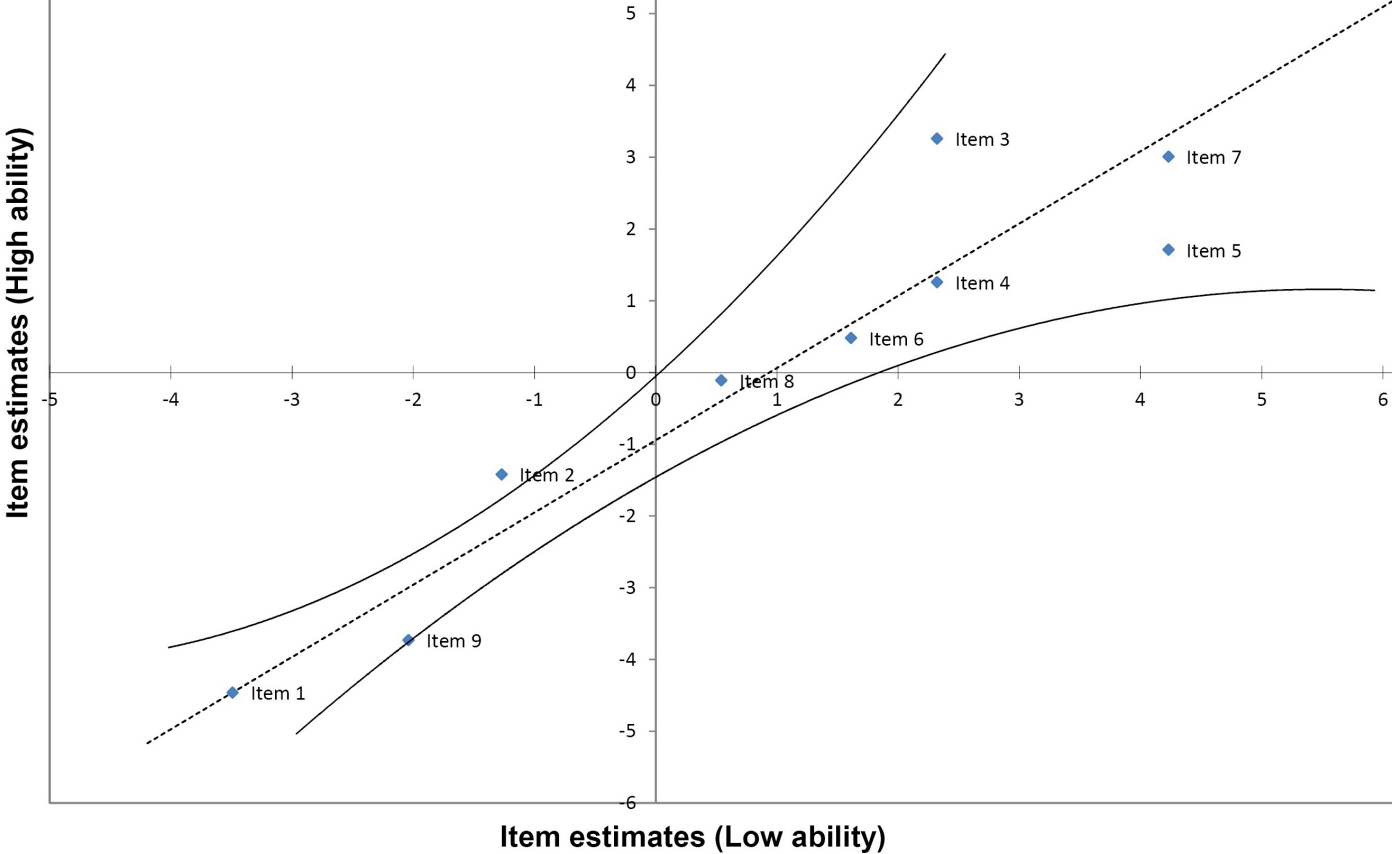
**Item difficulty invariance**.

The total sample of 351 MSM was divided into two subsamples. One contained those with raw scores of 0-2 (Low), and the other contained those who had raw scores above 3 (High). Data from each subsample were analyzed separately and the 9 item estimates (and SEs) for each group were imported into an Excel spreadsheet [7]. In figure 2, each black point represents an item. The curved lines indicate the 95% confidence intervals. The plot of item values showed that, with the notable exception of item 2, the item estimates of the risk behavior items were invariant (within error).

### Conversion of raw composition scores to logit scores

Table 3 presents raw composite scores and converted Rasch measurement scores. The raw composite scores are the sum of item scores (each item with a value of 0 or 1) and scores at the ordinal scale. The Rasch measures were estimated in the Rasch model, i.e., the logits. Each possible raw composition score (0-9) has a corresponding Rasch measure in the logit unit and standard error. The corresponding Rasch measures are at the interval scale.

**Table 3:** Conversion of raw composite scores to Rasch measure scores

Raw composite score	Rasch measure score	Standard error
0	-5.71	1.99
1	-4.02	1.32
2	-2.53	1.16
3	-1.26	1.08
4	-0.19	0.99
5	0.73	0.93
6	1.58	0.93
7	2.47	0.97
8	3.58	1.17
9	5.03	1.91

## Discussion

This study illustrates the utility of Rasch modeling for measuring the level of sexual risk for HIV/STI infection as a single latent construct and for establishing the relative degree of severity of each type of sexual behavior in HIV/STI transmission and acquisition. The Rasch model satisfactorily met the unidimensionality and local independence assumptions, had high reliable item reliability indices, acceptable item difficulty invariance and infit and outfit values. The HIV/STI-related risk behavior scale assesses a broad range of sexual behaviors including unprotected intercourse, commercial sex and concurrent sexual partnerships among MSM. The scale is suitable for use to measure the level of HIV/STI-related risk behaviors in countries where HIV/STI infection is becoming a critical problem among MSM. Because the measurement scale is at the interval level, it provides a useful measurement tool to inform, design and evaluate HIV/STI interventions that target behaviors.

The resulting Rasch scale provides a continuous measure from less to more extreme behaviors. Not surprisingly, two items, "had unsafe sex with female sex workers" and "had unsafe anal sex with male sex workers", have higher levels of item difficulty. Since engagement in the two types of sexual behaviors involves either male sex workers or female sex workers, those who have unprotected sex with these sex workers have a high degree of likelihood of HIV/STI acquisition and transmission. Previous studies have documented that sex workers are a core-transmitter group among MSM [22,23]. Although the proportions of the study subjects who reported these two high risky sexual behaviors are low in our study, they were substantial in previous studies [24,25]. In analyzing correlates of sexual risk, the Rasch measure can be directly used in univariate analysis and multiple modeling analyses since the Rasch measure scores are at the interval scale.

There are limitations in this study that should be noted. Because the study participants were recruited from one city, the sample was not representative of all areas in China. Future large scale studies need to be done to verify findings from this study. We used RDS to recruit MSM in our study. Because modeling techniques for analyzing RDS data are still under development, we could only use the unadjusted data in the Rasch model. Findings from this study may not be representative of MSM in general in China. Future studies need to assess the generalizability of our findings in more detail. The measurement scale included only 9 types of sexual behaviors, which has a limited coverage of the large domain of sexual risk. An item pool that draws from multiple measures of sexual behaviors may provide more complete and diversified coverage of the continuum of sexual risk for HIV/STI infection. Furthermore, the person reliability in our study was low at 0.35. This value reflected the mismatch of HIV/STI sexual risk behavior items to the level of HIV/STI sexual risk behaviors reported in the sample. However, based on the characteristics of the Rasch model (sample independency of item and item independency of person ability), analysis of the scale is not influenced. Another limitation of this study was the use of self-reported measures. Despite the anonymity of the survey, the face-to-face mode of the survey may have resulted in underreporting of sexual risk behaviors.

## Conclusions

The findings suggest that the Rasch model can be utilized for measuring the level of sexual risk for HIV/STI infection as a single latent construct and for establishing the relative degree of severity of each type of sexual behavior in HIV/STI transmission and acquisition among MSM. The measurement scale provides sources of reference in developing a scale that is normed on MSM in China. It also provides a useful measurement tool to inform, design and evaluate HIV/STI interventions focusing on behavioral aspects, which can be a valuable resource in the assessment of sexual behaviors among MSM in China and other similar countries.

## List of abbreviations used

MSM: Men who have sex with men; RDS: Respondent-driven sampling; PCA: Principle Components Analysis; MBs: Money boys.

## Competing interests

This research was supported partly by a research grant (107010-41-RGAT) from the Foundation for AIDS Research (amfAR), and partly by the Multidisciplinary HIV and TB Implementation Sciences Training in China funded by the US National Institutes of Health, Fogarty International Center and the National Institute on Drug Abuse with NIH Research Grant #U2R TW06918. All funders had no further role in study design; in the collection, analysis and interpretation of data; in the writing of the report; or in the decision to submit the paper for publication. All authors declare that they have no conflicts of interest.

## Authors' contributions

HL, TF and YC designed the study and wrote the protocol. JL and HL managed the literature searches and summaries of previous related work. Authors JL and HL undertook the statistical analysis and wrote the manuscript. All authors contributed to and have approved the final manuscript.

## Pre-publication history

The pre-publication history for this paper can be accessed here:

http://www.biomedcentral.com/1471-2458/11/763/prepub
